# Three new spider species of the genus *Pholcus* from the Taihang Mountains of China (Araneae, Pholcidae)

**DOI:** 10.3897/zookeys.600.7924

**Published:** 2016-06-22

**Authors:** Bao-Shi Zhang, Feng Zhang, Jing-Ze Liu

**Affiliations:** 1 College of Life Sciences, Hebei Normal University, Shijiazhuang, Hebei 050024, P. R. China; 2 Department of Biochemistry, Baoding University, Baoding, 071051, P. R. China; 3 The Key Laboratory of Invertebrate Systematics and Application, College of Life Sciences, Hebei University, Baoding, Hebei 071002, P. R. China

**Keywords:** Hebei Province, Pholcinae, Pholcus phungiformes, species group, taxonomy

## Abstract

In this study, three new species belonging to the genus *Pholcus*, collected from a forest of the Taihang Mountains, P. R. China, are described under the names of *Pholcus papillatus* **sp. n.** (male, female), *Pholcus curvus* **sp. n.** (male, female) and *Pholcus auricularis* **sp. n.** (male, female).

## Introduction

The spider family Pholcidae C. L. Koch, 1850 is the ninth largest spider family and, to date, 1461 species, belonging to 79 genera, have been reported (World Spider Catalog 2016). It has a worldwide distribution, but the highest diversity is concentrated in the tropical and subtropical regions. Pholcine spiders occupy a wide range of habitats in a variety of ecosystems, e.g., in buildings, under rocks, in caves, in leaf litter, and under leaves (Huber 2005a). Huber (2000, 2001, 2003a, b, c, 2005b, c, 2009a, 2011b) reported a large number of new species and revised many genera in this family. Based on cladistic analyses of morphological and molecular data and on qualitative character assessment (Huber 2011a; Dimitrov et al. 2013), Pholcidae was divided into five subfamilies: Arteminae Simon, 1893, Modisiminae Simon, 1893, Ninetinae Simon, 1890, Pholcinae C.L. Koch, 1850 and Smeringopinae Simon, 1893; Pholcinae is the most species-rich subfamily.

*Pholcus* Walckenaer, 1805 is the largest genus in Pholcinae, with 318 described species which are mainly distributed in the Old World. *Pholcus* can be diagnosed by the following combination of characters: male chelicerae usually with three pairs of apophyses; bulb usually with uncus and appendix; epigynum usually strongly sclerotized and with ‘knob’ (Huber 2011b). These *Pholcus* spiders frequently live in houses, rock-crevices, caves and leaf litter, and most spin loose and irregular webs in sheltered areas. Recently, the genus has been studied by several scholars: Huber (2001) made a few taxonomic remarks; Dimitrov and Ribera (2007) and Dimitrov et al. (2008) revised and cladistically analyzed the Macaronesian *Pholcus* species; Zhang and Zhu (2009) reviewed 55 Chinese *Pholcus* species; Huber (2011b) revised the genus *Pholcus* and presented 254 species in 29 species groups; Yao and Li (2012) described 35 new species and also provided new illustrations for 45 known species from China and two species from Laos; Yao and Li (2013) described two new species from Laos; Yao, Pham and Li (2015) described five new species from Vietnam.

The spider genus *Pholcus* Walckenaer, 1805 exhibits a highly diversity in China. One hundred fifteen *Pholcus* species attached to nine species groups (*Pholcus halabala* species group, *Pholcus ponticus* species group, *Pholcus crypticolens* species group, *Pholcus zham* species group, *Pholcus bidentatus* species group, *Pholcus nagasakiensis* species group, *Pholcus yichengicus* species group, *Pholcus taishan* species group, and *Pholcus phungiformes* species group) were recorded from China (Zhang and Zhu 2009; Tong and Ji 2010; Tong and Li 2010; Huber 2011b; Peng and Zhang 2011a, b; Yao and Li 2012; Liu and Tong 2015; World Spider Catalog 2016).

The Taihang Mountains are located in the northeastern China, between 34°34'N to 40°43'N and 110°14'E to 114°33'E. Fuping county, a county of Hebei Province, is located in the Taihang Mountains area. To explore the diversity of the arthropod in Fuping county, one survey was carried out in 2014. While examining the spider specimens collected from leaf litter in this survey, three new species belonging to *Pholcus* were found and are reported in the present paper. Detailed diagnosis, descriptions, and illustrations of these new taxa are presented.

## Material and methods

All specimens were preserved in 75% ethanol and examined, drawn, and measured under a Nikon SMZ1500 stereomicroscope equipped with a drawing tube. Photographs were taken with a Leica M205A stereomicroscope equipped with a Leica DFC550 Camera and LAS software (Ver. 4.6). Male and female genitalia were examined and illustrated after dissection. Epigyna were removed and treated in 10% warm solution of potassium hydroxide (KOH) before illustration. Left pedipalpi of male spiders were illustrated, except as otherwise indicated. All measurements are given in millimeters. Eye sizes were measured as the maximum diameter of the lens in dorsal or frontal view. Leg measurements are given as total length (femur + patella + tibia + metatarsus + tarsus). Leg segments were measured on their dorsal side. Terminology and taxonomic descriptions follow Huber (2000, 2009b).

The following abbreviations are used in the text:

AER
 anterior eye row 


ALE
 anterior lateral eye 


AME
 anterior median eye 


MOA
 median ocular area 


PER
 posterior eye row 


PLE
 posterior lateral eye 


PME
 posterior median eye 


b
 bulb 


e
 embolus 


pa
 pseudo-appendix 


pp
 pore plate 


pr
 procursus 


u
 uncus 


All specimens used in this studied are deposited in the Museum of Hebei University, Baoding, P. R. China (MHBU).

## Taxonomy

### Family Pholcidae C. L. Koch, 1850

#### Genus Pholcus

Taxon classificationAnimaliaAraneaePholcidae

Walckenaer, 1805

##### Type species.

*Pholcus phalangioides* (Fuesslin, 1775)

### *Pholcus phungiformes* species group

The *Pholcus phungiformes* group is largely distributed in northeastern China and the Korean Peninsula. Most species of this group have the following characters: carapace with vivid pattern, abdomen cylindrical, male chelicerae with proximal apophyses frontally, male palpal tibia with prolatero-ventral modification, procursus with dorsal spines, appendix absent, sometimes with pseudo-appendix (apophysis arising from uncus rather than from bulb, near usual position of appendix), epigynum sclerotized, with knob-shaped apophysis (Huber 2011). The pseudo-appendix of *Pholcus exilis* auct, date, *Pholcus wuling* auct, date, and *Pholcus chicheng* auct, date, may be bifid, but the character needs further study. The three new *Pholcus* species are assigned as members of this group in possessing most of the characters of the *Pholcus phungiformes* group.

#### Pholcus papillatus sp. n.

Taxon classificationAnimaliaAraneaePholcidae

http://zoobank.org/3557D5D5-09B8-4733-BE13-767A8078CB1C

Figs 1 , 2 , 3 , 4

##### Type material.

**Holotype**: male (MHBU), CHINA: Hebei Province, Fuping County, Longquanguan Town, Liaodaobei Village, 38°16'N, 114°17'E, alt. 1700 m, 6 August 2014, B.S. Zhang leg. **Paratypes**: 1 male and 3 females (MHBU), same data as in holotype.

##### Etymology.

The specific name is from Latin word “*papillatus*”, in reference to the shape of epigynal apophysis; adjective.

##### Diagnosis.

**Figure 1.**
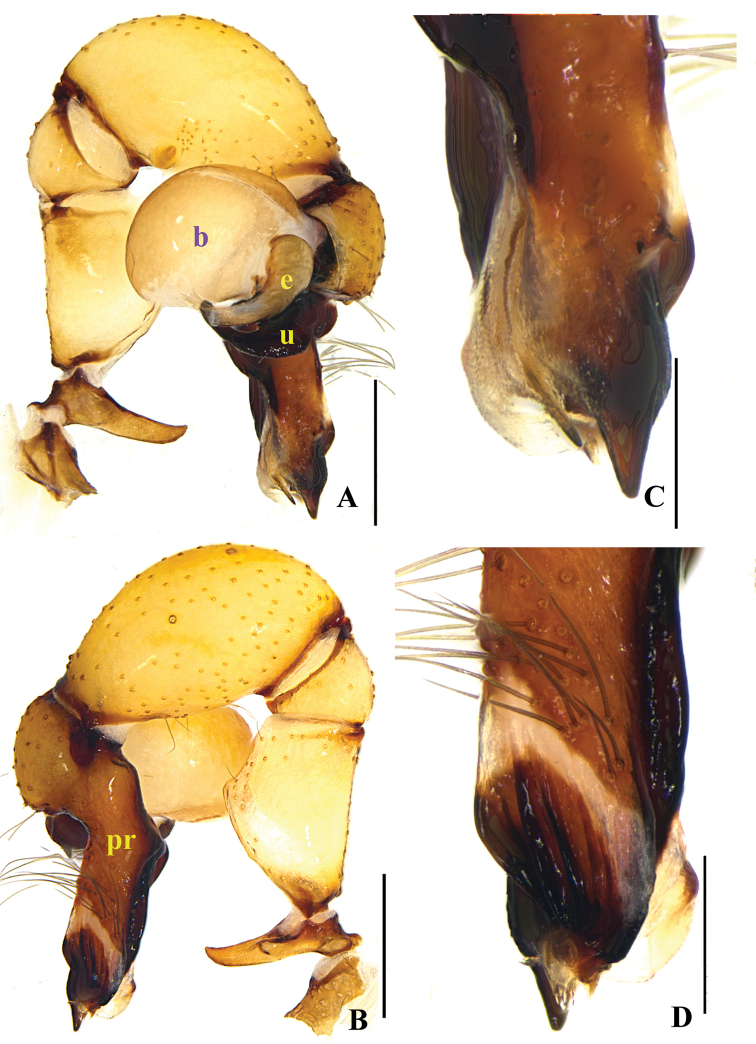
*Pholcus papillatus* sp. n., male holotype. **A–B** Pedipalpus (**A** prolateral view **B** retrolateral view) **C–D** Distal part of procursus (**C** prolateral view **D** retrolateral view). Scale bars: 0.2 mm (**C, D**); 0.5 mm (**A, B**).

**Figure 2.**
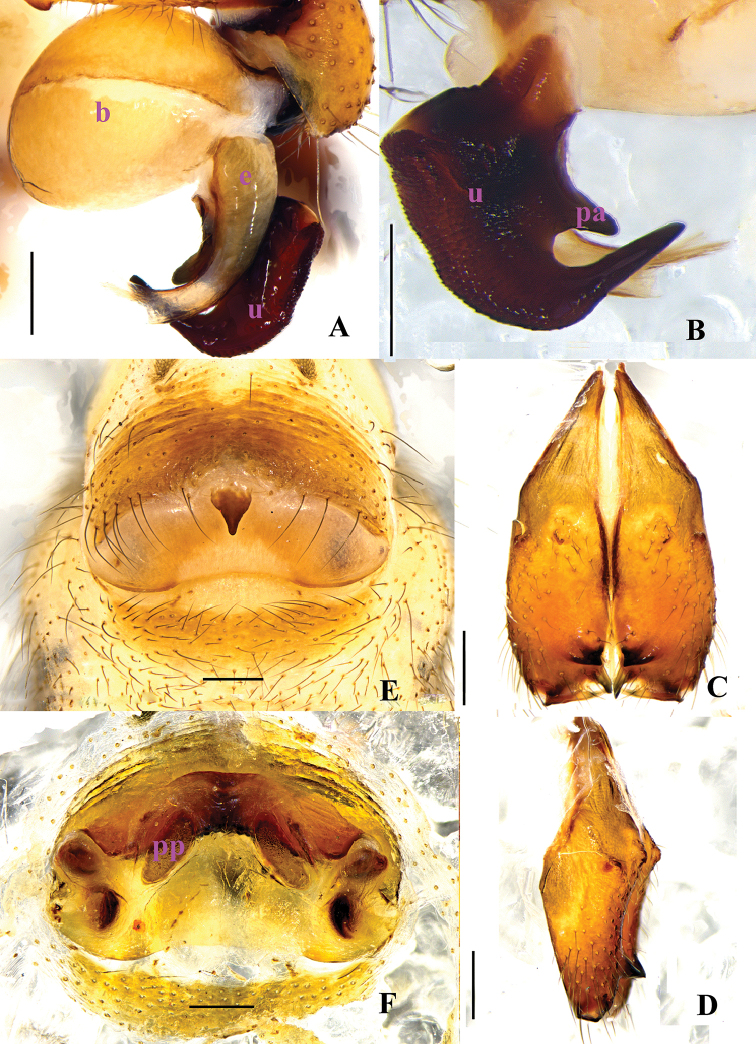
*Pholcus papillatus* sp. n., male holotype (**A–D**) and female paratype (**E–F**). **A–B** Bulb and uncus (**A** prolateral view **B** retrolateral view) **C–D** Chelicerae (**C** frontal view **D** lateral view) **E** Epigynum, ventral view **F** Vulva, dorsal view. Scale bars: 0.2 mm.

**Figure 3.**
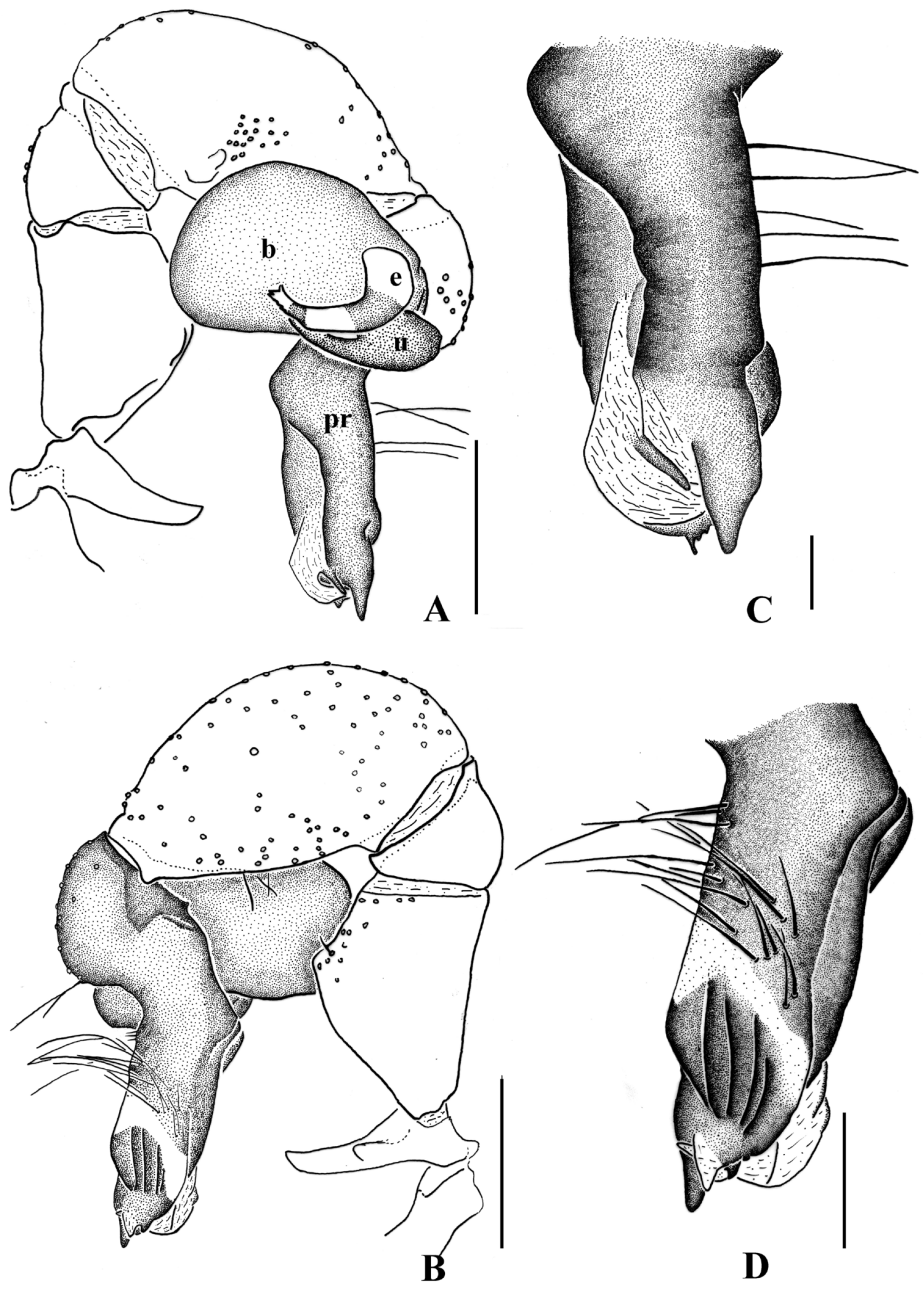
*Pholcus papillatus* sp. n., male holotype. **A–B** Pedipalpus (**A** prolateral view **B** retrolateral view) **C–D** Distal part of procursus (**C** prolateral view **D** retrolateral view). Scale bars: 0.2 mm.

**Figure 4.**
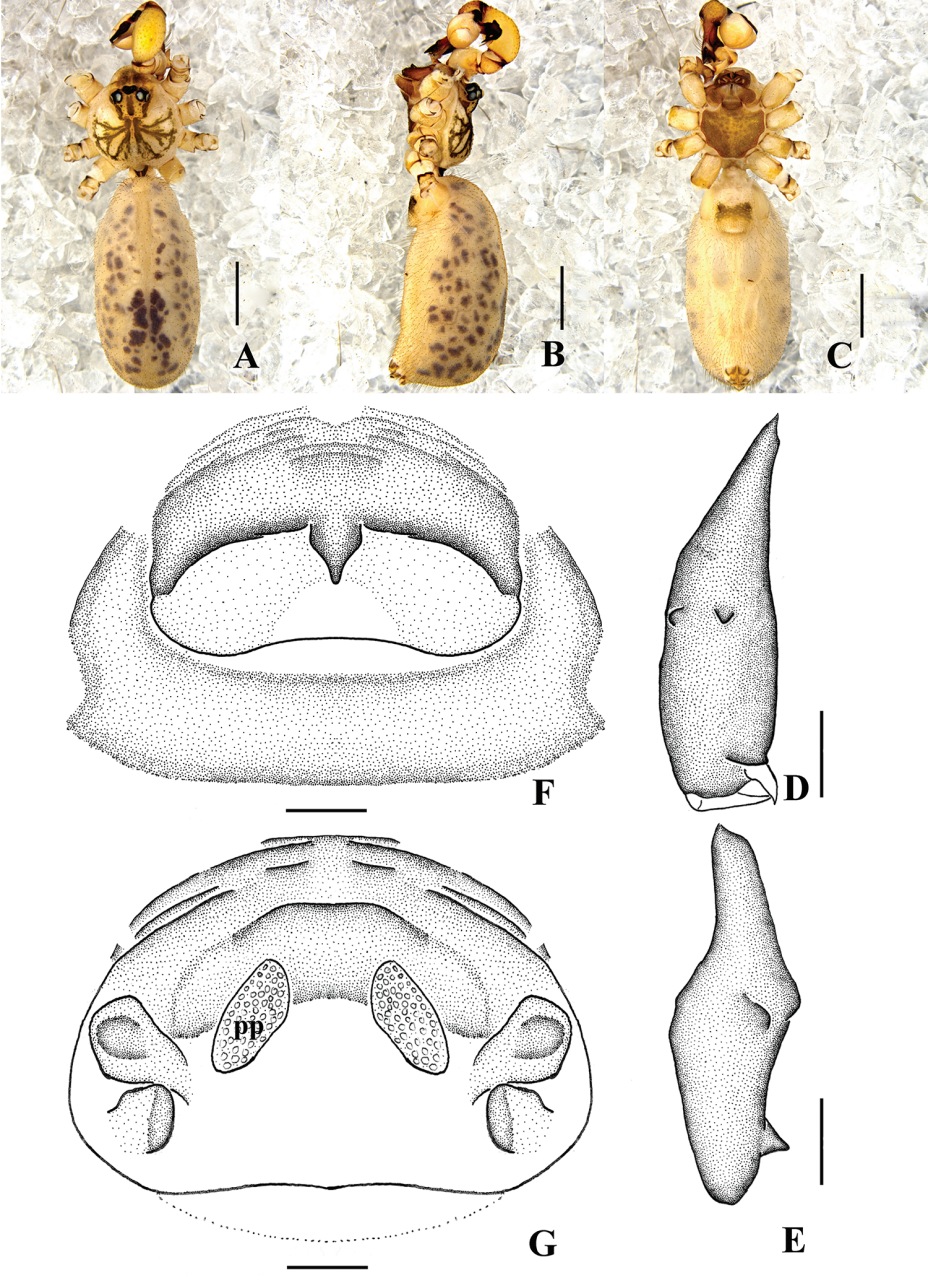
*Pholcus papillatus* sp. n., male holotype (**A–E**) and female paratype (**F–G**). **A–C** Habitus (**A** dorsal view **B** lateral view **C** ventral view) **D–E** Chelicerae (D, frontal view **E** lateral view) **F** Epigynum, ventral view **G** Vulva, dorsal view. Scale bars: 0.2 mm (**D–G**); 1.0 mm (**A–C**).

Narrow, long pseudo-appendix originating from the uncus (Fig. 2B). Distinguished from similar species with a pseudo-appendix by: palpal bulb with longer uncus, procursus with one spine-shaped projection and one hook-shaped membranous projection on tip (Figs 1A–D, 2A–B, 3A–D). The females of the new species are distinguished from females of similar species by the larger teat-shaped epigynal apophysis (Figs 2E, 4F).

##### Description.

**Male** (holotype): Total length 4.90 (5.10 with clypeus), prosoma 1.40 long, 1.52 wide, opisthosoma 3.36 long, 1.68 wide. Habitus as in Fig. 4A–C. Dorsal shield of prosoma pale grey, with dark brown radiated stripes and bands marginally; thoracic groove distinct; ocular area elevated, with short eye-stalks; ocular area yellow-brown, with a median dark brown band and two lateral dark brown bands beside PLEs, dorsal prosoma and ocular area both with dispersed dark brown spots, of them, two distinct spots behind PMEs; clypeus 0.23 high, dark brown, with light margin. Diameter AME 0.08, ALE 0.13, PME 0.12, PLE 0.13. Distance AME-AME 0.07, AME-ALE 0.10, PME-PME 0.24, PME-PLE 0.04, ALE-ALE 0.62, PLE-PLE 0.78. MOA 0.23 long, front width 0.23, back width 0.46. Chelicerae as in Figs 2C–D and 4D–E, with pair of black apophyses distally, pair of unsclerotized, small and nearly nipple-shaped apophyses proximolaterally and frontally. Labium and endites dark brown, distal part pale, labium wider than long (0.32/0.15). Sternum wider than long (1.04/0.77), dark brown, median part of sternum light. Legs long, brown, but dark brown on proximal parts of tibiae, and on distal parts of femora and tibiae, with whitish on subdistal parts of femora and tibiae. Measurements of legs: I 36.78 (9.59 + 0.59 + 9.69 + 15.43 + 1.48), II 26.81 (7.37 + 0.55 + 5.89 + 11.61 + 1.39), III 22.46 (6.55 + 0.61 + 5.42 + 8.59 + 1.29), IV 24.58 (7.06 + 0.53 + 5.74 + 9.89 + 1.36); tibia I L/d: 62. Leg formula: 1243. Opisthosoma pale grey, with dark spots dorsally and laterally. Pedipalpi illustrated in Figs 1A–D and 3A–D; trochanter with a long ventral apophysis; tibia with a small projection prolaterally; procursus simple proximally and complex distally, dorsal spines present; uncus with a pseudo-appendix, the tip of uncus long and bent; embolus weakly sclerotized.

**Female.** Generaly similar to male. One specimen measured: total length 4.58 (4.80 with clypeus), prosoma 1.34 long, 1.46 wide, opisthosoma 3.09 long, 1.44 wide. clypeus 0.23 high. Diameter AME 0.06, ALE 0.10, PME 0.09, PLE 0.11. Distance AME-AME 0.08, AME-ALE 0.07, PME-PME 0.19, PME-PLE 0.04, ALE-ALE 0.50, PLE-PLE 0.64. MOA 0.24 long, front width 0.18, back width 0.39. labium wider than long (0.31/0.18). Sternum wider than long (1.00/0.74). Measurements of legs: I 36.60 (9.55 + 0.58 + 9.59 + 15.41 + 1.47), II 26.66 (7.33 + 0.54 + 5.88 + 11.54 + 1.37), III 22.32 (6.51 + 0.58 + 5.38 + 8.58 + 1.27), IV 24.49 (7.01 + 0.52 + 5.72 + 9.88 + 1.36); tibia I L/d: 60. Leg formula: 1243. Epigynum (Figs 2E and 4F) brown, roughly triangular, with distinct patterns and a teat-shaped apophysis on the top. Dorsal view of vulva (Figs 2F and 4G) with a rainbow-shaped, sclerotized arch anteriorly and two long ovoid pore plates, and a nearly meniscate sclerite.

##### Variation.

Male: Total body length: 4.58, 4.90. Tibia I (n = 2): 8.96, 9.59 (mean: 9.28). Female: Total body length: 4.58, 4.63, 4.81. Tibia I (n = 3): 9.55, 9.70, 9.78 (mean 9.68).

##### Distribution.

Only known from the type locality.

##### Remarks.

The females also resemble *Pholcus foliaceus* Peng & Zhang, 2013, but can be distinguished by the precurved margin of anterior plate of the epigynum and the long ovoid pore plates (Figs 2E–F, 4F–G).

#### Pholcus curvus sp. n.

Taxon classificationAnimaliaAraneaePholcidae

http://zoobank.org/81E106FA-EB6F-4ACB-89E8-1ECD43AD0E3C

Figs 5 , 6 , 7 , 8

##### Type material.

**Holotype**: male (MHBU), CHINA: Hebei Province, Fuping County, Longquanguan Town, Heiyagou Village, 38°16'N, 114°05'E, alt. 900 m, 5 August 2014, B.S. Zhang leg. **Paratypes**: 2 males and 8 females (MHBU), same data as in holotype.

##### Etymology.

The specific name is from the Latin word “*curvus*”, in reference to the shape of the palpal uncus; adjective.

##### Diagnosis.

**Figure 5.**
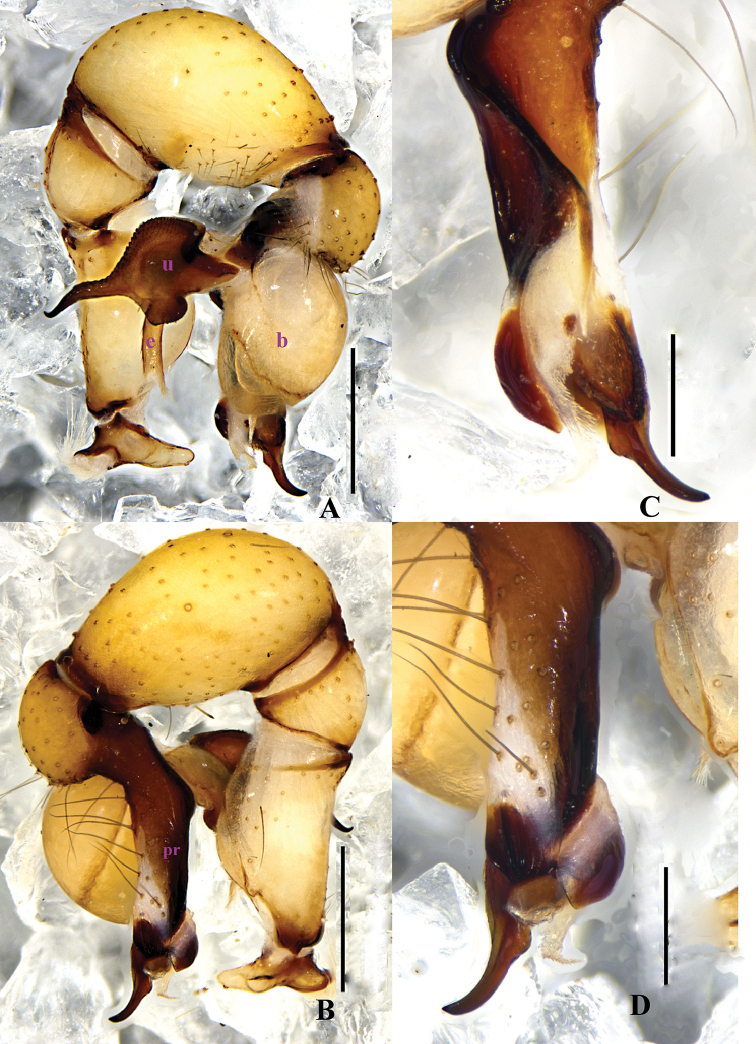
*Pholcus curvus* sp. n., male holotype. **A–B** Pedipalpus (**A** prolateral view **B** retrolateral view) **C–D** Distal part of procursus (**C** prolateral view **D** retrolateral view). Scale bars: 0.2 mm.

**Figure 6.**
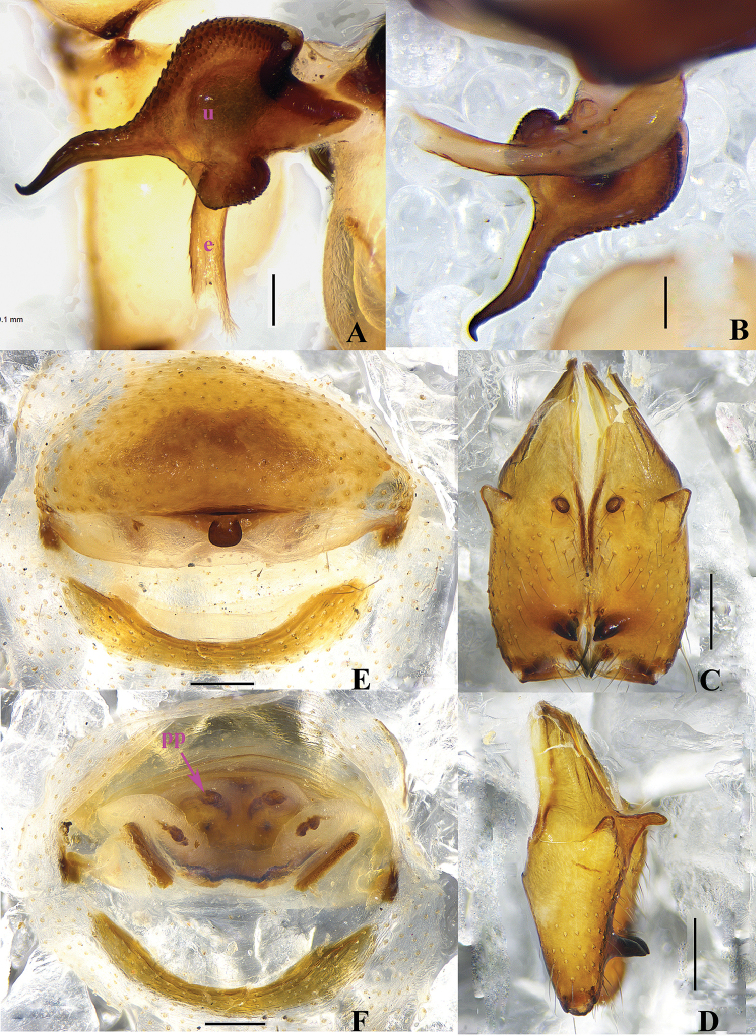
*Pholcus curvus* sp. n., male holotype (**A–D**) and female paratype (**E–F**). **A–B** Bulb and uncus (**A** prolateral view **B** retrolateral view) **C–D** Chelicerae (**C** frontal view **D** lateral view) **E** Epigynum, ventral view **F** Vulva, dorsal view. Scale bars: 0.1 mm (**A, B**); 0.2 mm (**C–F**).

**Figure 7.**
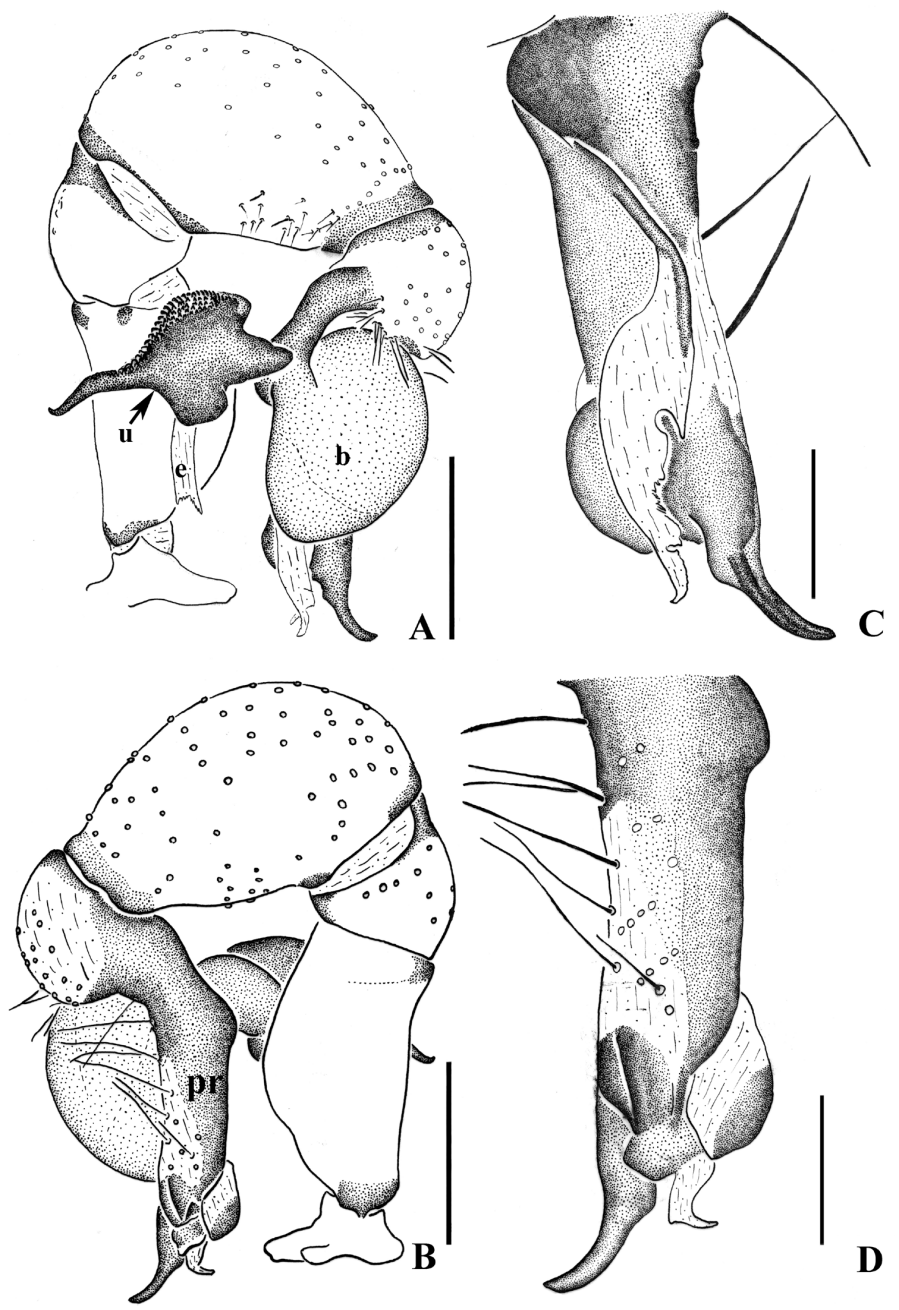
*Pholcus curvus* sp. n., male holotype. **A–B** Pedipalpus (**A** prolateral view **B** retrolateral view) **C–D** Distal part of procursus (**C** prolateral view **D** retrolateral view). Scale bars: 0.2 mm.

**Figure 8.**
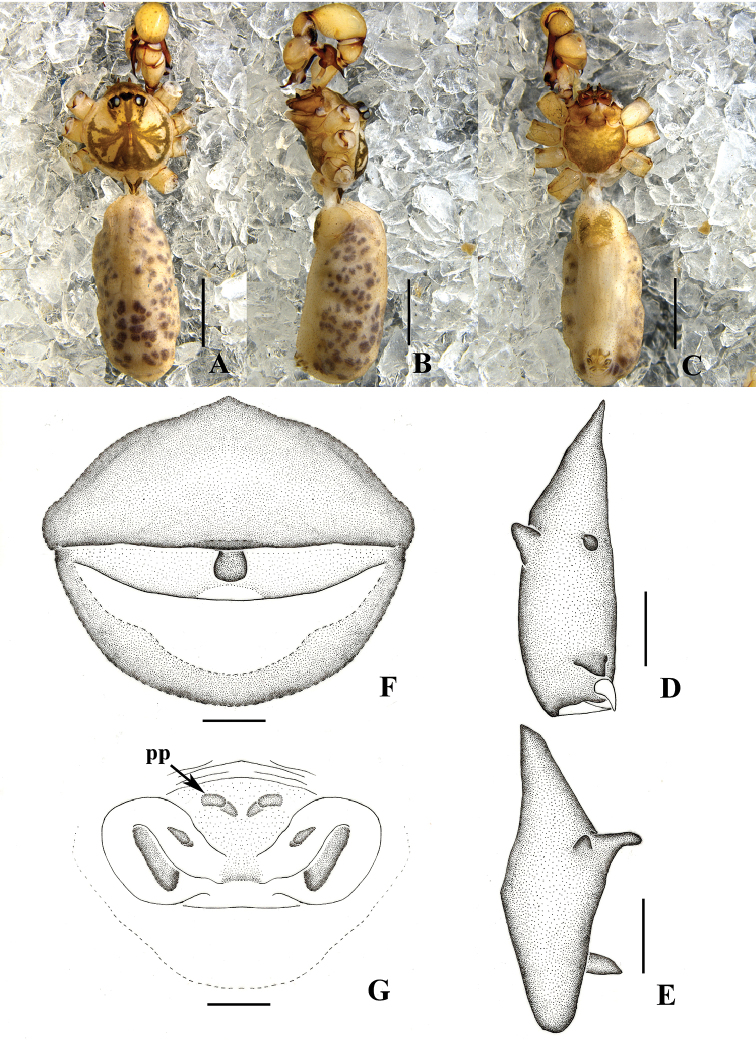
*Pholcus curvus* sp. n., male holotype (**A–E**) and female paratype (**F–G**). **A–C** Habitus (**A** dorsal view **B** lateral view **C** ventral view) **D–E** Chelicerae (**D** frontal view **E** lateral view) **F** Epigynum, ventral view **G** Vulva, dorsal view. Scale bars: 0.2 mm (**D–G**); 1.0 mm (**A–C**).

Distinguished by the S-shaped tip of uncus, the long and curve beak-shaped tip of the procursus, the hat-shaped membranous projection near tip, the small and almost quadrate-shaped epigynal apophysis (Figs 5A–D, 7A–D, 6E, 8F).

##### Description.

Male (holotype): Total length 4.50 (4.63 with clypeus), prosoma 1.36 long, 1.41 wide, opisthosoma 2.85 long, 1.24 wide. Habitus as in Fig. 8A–C. Dorsal shield of prosoma pale grey, with brown radiated stripes and bands marginally; thoracic groove distinct; ocular area elevated, with short eye-stalks; ocular area yellow-brown, with a median brown band and two lateral brown bands beside PLEs, dorsal prosoma and ocular area both with dispersed brown spots, of them, two distinct spots behind PMEs; clypeus 0.20 high, brown. Diameter AME 0.07, ALE 0.08, PME 0.15, PLE 0.13. Distance AME-AME 0.06, AME-ALE 0.05, PME-PME 0.24, PME-PLE 0.06, ALE-ALE 0.49, PLE-PLE 0.62. MOA 0.24 long, front width 0.22, back width 0.42. Chelicerae as in Figs 6C–D and 8D–E, with pair of black apophyses, pair of unsclerotized thumb-shaped apophyses proximolaterally, and pair of nearly long finger-shaped apophyses frontally. Labium and endites brown, distal part pale, labium wider than long (0.32/0.17). Sternum wider than long (1.00/0.72), brown, median part of sternum light. Legs long, brown, with two dark brown ring spots on proximal parts of femora and four dark brown ring spots on tibiae. Measurements of legs: I 33.98 (9.57 + 0.53 + 9.23 + 13.23 + 1.42), II 24.63 (6.96 + 0.58 + 6.30 + 9.49 + 1.30), III 17.75 (5.22 + 0.41 + 4.32 + 6.79 + 1.01), IV 23.52 (6.90 + 0.47 + 5.89 + 9.16 + 1.10); tibia I L/d: 60. Leg formula: 1243. Opisthosoma pale grey, with dark spots dorsally and laterally. Pedipalpi as in Figs 5A–D and 7A–D; trochanter with a moderate ventral apophysis; proximal femur with a small apophysis retrolaterally; tibia with a sheet-shaped projection prolaterally; procursus simple and tip with a narrow process, dorsal spines present; uncus long and bent, with a scaly edge; embolus weakly sclerotized.

**Female.** Generaly similar to the male. One specimens measured: total length 5.09 (5.26 with clypeus), prosoma 1.27 long, 1.52 wide, opisthosoma 3.59 long, 1.61 wide, clypeus 0.20 high. Diameter AME 0.08, ALE 0.10, PME 0.13, PLE 0.12. Distance AME-AME 0.07, AME-ALE 0.07, PME-PME 0.17, PME-PLE 0.05, ALE-ALE 0.45, PLE-PLE 0.59. MOA 0.25 long, front width 0.14, back width 0.36. Labium wider than long (0.31/0.17). Sternum wider than long (1.05/0.79). Measurements of legs: I 32.15 (7.99 + 0.55 + 8.09 + 13.42 + 2.10), II 20.24 (6.31 + 0.42 + 5.60 + 6.55 + 1.36), III 17.40 (4.71 + 0.55 + 3.87 + 6.95 + 1.32), IV 20.12 (6.03 + 0.64 + 5.53 + 6.55 + 1.37); tibia I L/d: 50. Leg formula: 1243. Epigynum (Figs 6E and 8F) brown, roughly striped with distinct patterns and an almost quadrate-shaped apophysis on the top. Dorsal view of vulva (Figs 6F and 8G) with a rainbow-shaped, sclerotized arch anteriorly, two bent pore plates, and two long and bent sclerites.

##### Variation.

Male: Total body length: 4.38, 4.43, 4.50. Tibia I (n = 3): 9.36, 9.46, 9.57 (mean: 9.46). Female: Total body length 4.78–5.09. Tibia I (n = 8): 7.65–7.99 (mean 7.81).

##### Distribution.

Only known from the type locality.

##### Remarks.

Among the *Pholcus phungiformes* group, the males of the new species resemble *Pholcus hamatus* Tong & Ji, 2010 by the following: uncus with a narrow, long and bent tip and a robust apophysis, without appendix and pseudo-appendix (Figs 5A, 6A–B, 7A). The females of the new species are distinguished from those of *Pholcus hamatus* by the small and almost quadrate-shaped epigynal apophysis (Figs 6E, 8F).

#### Pholcus auricularis sp. n.

Taxon classificationAnimaliaAraneaePholcidae

http://zoobank.org/294FFD7E-3826-48F3-8E79-A5BC1FA40825

**Figure 9.**
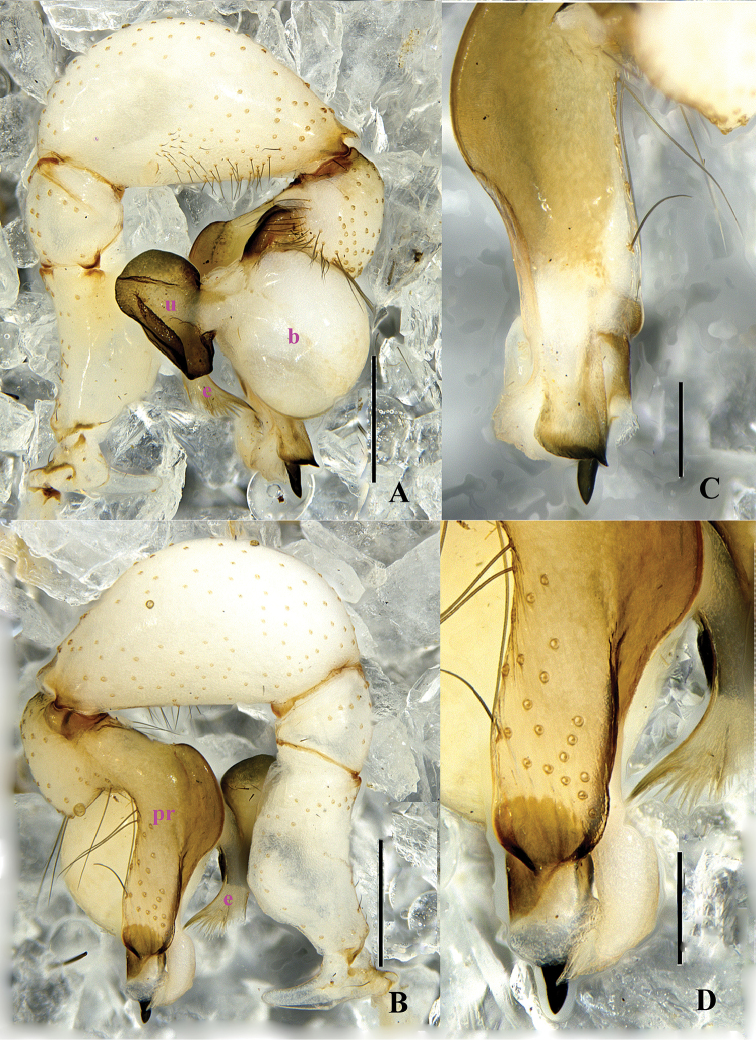
*Pholcus auricularis* sp. n., male holotype. **A–B** Pedipalpus (**A** prolateral view **B** retrolateral view) **C–D** Distal part of procursus (**C** prolateral view **D** retrolateral view). Scale bars: 0.2 mm (**C, D**); 0.5 mm (**A, B**).

**Figure 10.**
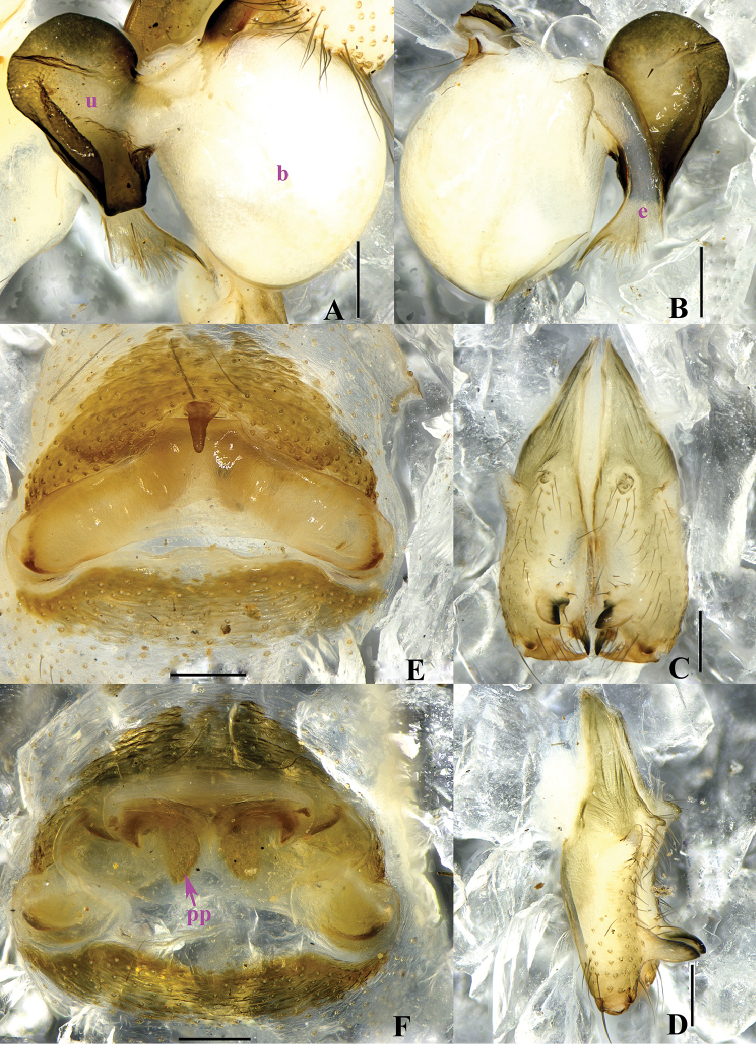
*Pholcus auricularis* sp. n., male holotype (**A–D**) and female paratype (**E–F**) **A–B** Bulb and uncus (**A** prolateral view **B** retrolateral view) **C–D** Chelicerae (**C** frontal view **D** lateral view) **E** Epigynum, ventral view **F** Vulva, dorsal view. Scale bars: 0.2 mm.

**Figure 11.**
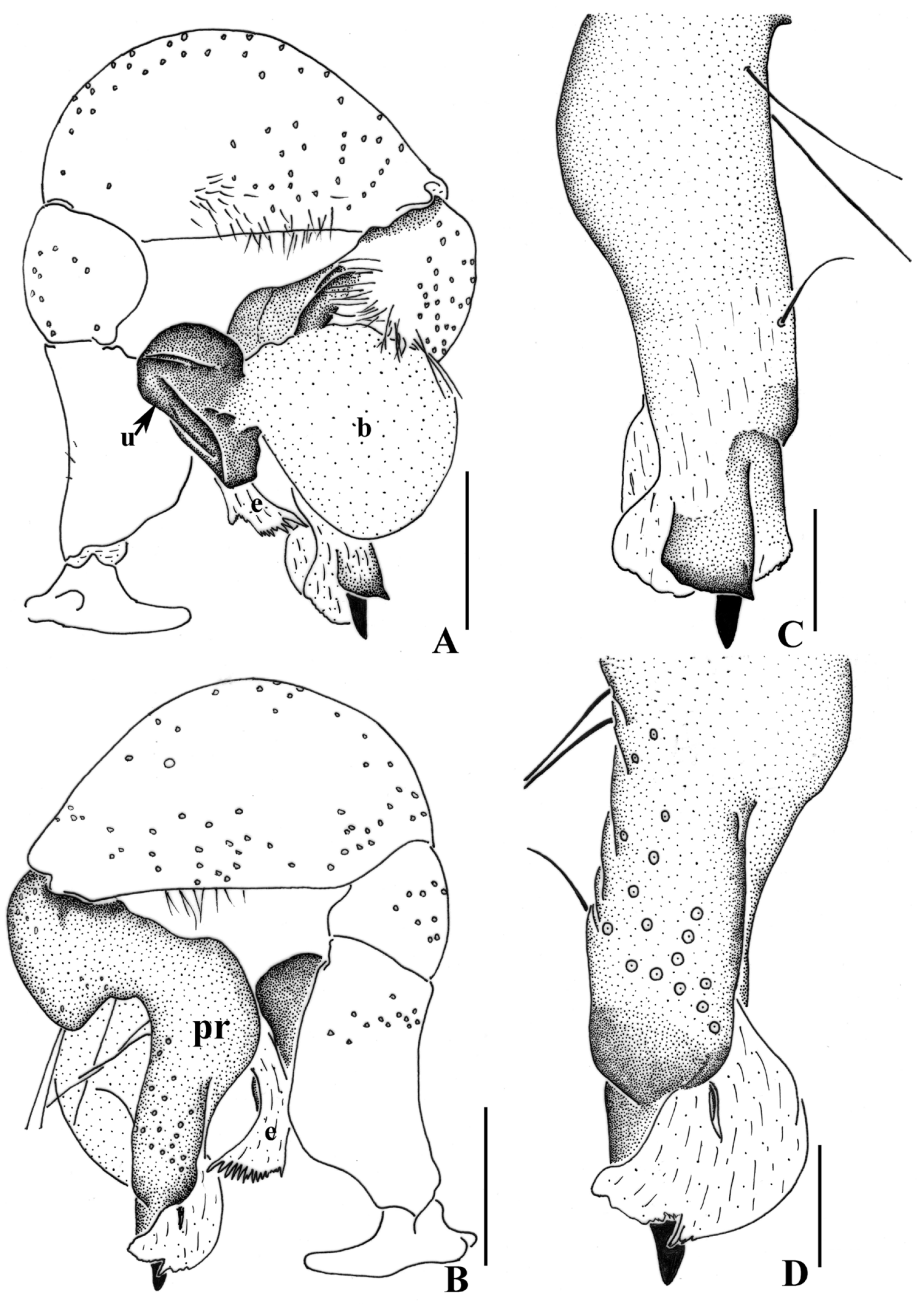
*Pholcus auricularis* sp. n., male holotype. **A–B** Pedipalpus (**A** prolateral view **B** retrolateral view) **C–D** Distal part of procursus (**C** prolateral view **D** retrolateral view). Scale bars: 0.2 mm (**C, D**); 0.5 mm (**A, B**).

**Figure 12.**
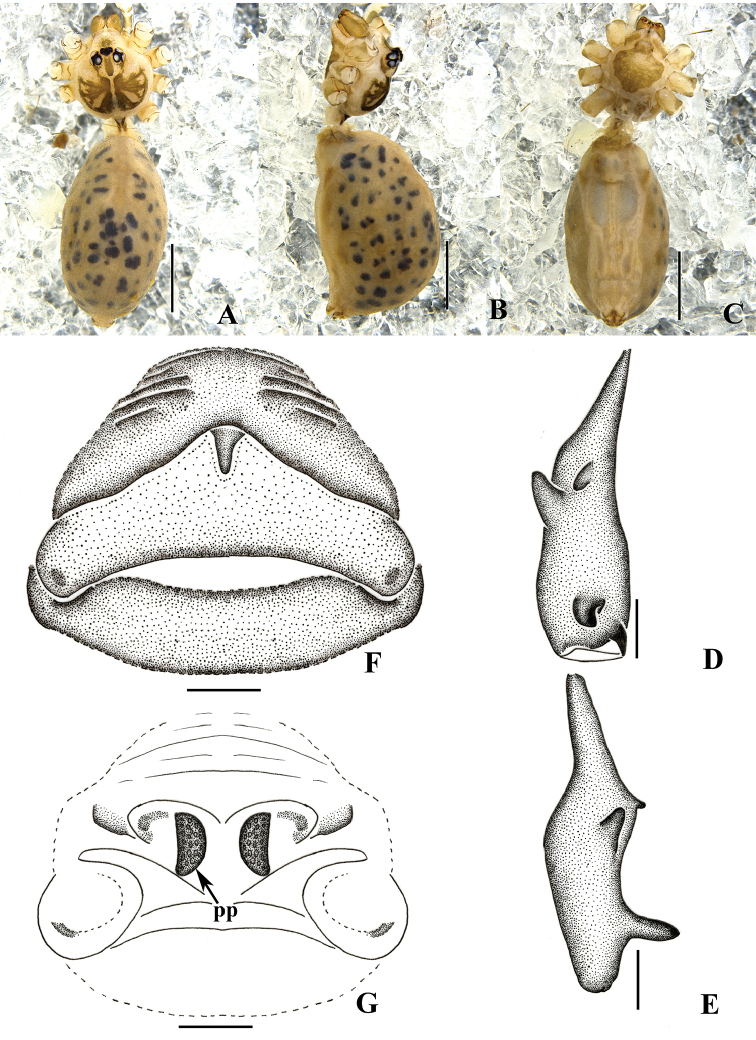
*Pholcus auricularis* sp. n., male holotype (**A–E**) and female paratype (**F–G**) **A–C** Habitus (**A** dorsal view **B** lateral view **C** ventral view) **D–E** Chelicerae (**D** frontal view **E** lateral view) **F** Epigynum, ventral view **G** Vulva, dorsal view. Scale bars: 0.2 mm (**D–G**); 1.0 mm (**A–C**).

Figs 9 , 10 , 11 , 12

##### Type material.

**Holotype**: male (MHBU), CHINA: Hebei Province, Fuping County, Longquanguan Town, Liaodaobei Village, 38°16'N, 114°17'E, alt. 1050 m, 5 August 2014, B.S. Zhang leg. **Paratypes**: 6 females (MHBU), same data as in holotype.

##### Etymology.

The specific name is from the Latin word “*auricularis*”, in reference to the shape of pedipalpi; adjective.

##### Diagnosis.

Distinguished by the combination of the following characters: uncus thin, ear-shaped, the tip of procursus thin and extending downward, epigynal apophysis short, thin and clavate, the tip thinner (Figs 9A–D, 10A–B, 10E, 11A–D, 12F).

##### Description.

Male (holotype): Total length 4.13 (5.32 with clypeus), prosoma 1.07 long, 1.19 wide, opisthosoma 2.87 long, 1.54 wide. Habitus as in Figs 12A–C. Dorsal shield of prosoma pale grey, with brown radiated stripes and bands marginally; thoracic groove absent; ocular area elevated, with short eye-stalks; ocular area pale grey, with a wide median brown band and two lateral brown bands beside PLEs, dorsal prosoma and ocular area both with dispersed dark brown spots, of them, two distinct spots behind PMEs; clypeus 0.21 high, pale grey, median part with brown patch. Diameter AME 0.07, ALE 0.10, PME 0.08, PLE 0.09. Distance AME-AME 0.04, AME-ALE 0.06, PME-PME 0.17, PME-PLE 0.06, ALE-ALE 0.47, PLE-PLE 0.54. MOA 0.24 long, front width 0.16, back width 0.35. Chelicerae as in Figs 10C-D and 12D-E, with pair of long apophyses distally and prolateral part black, pair of unsclerotized thumb-shaped apophyses proximolaterally, and pair of nearly nipple-shaped apophyses frontally. Labium and endites brown, distal part pale, labium wider than long (0.27/0.19). Sternum wider than long (0.73/0.58), brown, median part of sternum light, margin pale grey. Legs long, pale grey, with four brown ring spots on femora and tibiae, one brown ring spot on proximal parts of metatarsi. Measurements of legs: I 21.14 (5.61 + 0.44 + 5.61 + 7.87 + 1.61), II 15.13 (4.54 + 0.27 + 3.75 + 5.34 + 1.23), III 11.14 (3.15 + 0.34 + 2.72 + 3.90 + 1.03), IV 14.89 (4.22 + 0.30 + 3.69 + 5.28 + 1.40); tibia I L/d: 51. Leg formula: 1243. Opisthosoma pale grey, with dark spots dorsally and laterally. Pedipalpi as in Figs 9A–D and 11A–D; trochanter with a moderate ventral apophysis; tibia with a sheet-shaped projection prolaterally; procursus simple, its tip extending downward, dorsal spines absent; uncus and embolus weakly sclerotized.

**Female.** Similar to male. One specimen measured: total length 4.95 (5.12 with clypeus), prosoma 1.35 long, 1.48 wide, opisthosoma 3.42 long, 2.35 wide. clypeus 0.22 high, yellow. Diameter AME 0.04, ALE 0.10, PME 0.10, PLE 0.12. Distance AME-AME 0.07, AME-ALE 0.06, PME-PME 0.23, PME-PLE 0.06, ALE-ALE 0.50, PLE-PLE 0.61. MOA 0.21 long, front width 0.19, back width 0.37. Labium wider than long (0.27/0.18). Sternum wider than long (0.97/0.75). Measurements of legs: I 21.82 (7.00 + 0.56 + 5.59 + 7.42 + 1.25), II 18.03 (5.23 + 0.55 + 4.57 + 6.53 + 1.15), III 13.44 (3.80 + 0.39 + 3.31 + 4.91 + 1.03), IV 17.74 (4.86 + 0.52 + 4.48 + 6.69 + 1.19); tibia I L/d: 63. Leg formula: 1243. Epigynum (Figs 10E and 12F) brown, roughly triangular, with distinct patterns and a short rod-like apophysis on the top. Dorsal view of vulva (Figs 10F and 12G) with an M-shaped, sclerotized arch anteriorly, two closely spaced semilunar pore plates.

##### Variation.

Female: Total body length 4.68–4.95. Tibia I (n = 6): 6.56–7.00 (mean 6.86).

##### Distribution.

Only known from the type locality.

##### Remarks.

Among the *Pholcus phungiformes* group, the new species resembles *Pholcus alloctospilus* Zhu & Gong, 1991 and *Pholcus fengchen* Zhang & Zhu, 2009, but can be distinguished from those by: the short and thick club-shaped projection on tip of procursus, chitinized plate of the epigynum triangular arch (Figs 9C–D, 10E, 11C–D, 12F).

## Conclusions

The *Pholcus phungiformes* group includes 52 nominal species, of which 33 species were recorded from China (Zhang and Zhu 2009; Tong and Ji 2010; Tong and Li 2010; Huber 2011b; Chen, Zhang and Zhu 2011; Yao and Li 2012; Peng and Zhang 2013; Seo 2014; Liu and Tong 2015). Most of these chinese species are distributed in northeastern China: from Hebei Province *Pholcus alloctospilus*, *Pholcus pennatus*, *Pholcus zhuolu*, *Pholcus triangulates*, *Pholcus wangxidong*, *Pholcus chicheng*, *Pholcus datan*, *Pholcus babao*, *Pholcus wuling*, *Pholcus jinniu*, *Pholcus exilis*, *Pholcus papillatus* sp. n., *Pholcus curvus* sp. n. and *Pholcus auricularis* sp. n. are known, from Beijing municipality *Pholcus beijingensis*, *Pholcus brevis*, from Liaoning Province *Pholcus suizhongicus*, *Pholcus jiuwei*, *Pholcus fengcheng*, *Pholcus phoenixus*, *Pholcus gaoi*, *Pholcus wangtian*, *Pholcus tongi*, *Pholcus wangi*, *Pholcus decorus*, *Pholcus hamatus*, *Pholcus lingulatus*, *Pholcus foliaceus*, *Pholcus xianrendong* and *Pholcus sublingulatus*, from Shanxi Province *Pholcus luya*, and from both Hebei and Liaoning Provinces *Pholcus clavimaculatus*, only *Pholcus xingren* occurs in Guizhou Province.

## Supplementary Material

XML Treatment for Pholcus

XML Treatment for Pholcus papillatus

XML Treatment for Pholcus curvus

XML Treatment for Pholcus auricularis
